# Progesterone Receptor Signaling Selectively Modulates Cytokine-Induced Global Gene Expression in Human Cervical Stromal Cells

**DOI:** 10.3389/fgene.2020.00883

**Published:** 2020-09-11

**Authors:** Douglas A. Kniss, Taryn L. Summerfield

**Affiliations:** ^1^Division of Maternal-Fetal Medicine and Laboratory of Perinatal Research, Department of Obstetrics and Gynecology, The Ohio State University, College of Medicine and Wexner Medical Center, Columbus, OH, United States; ^2^Department of Biomedical Engineering, College of Engineering, The Ohio State University, Columbus, OH, United States

**Keywords:** cervix, inflammation, microRNA, preterm birth, transcriptomics

## Abstract

Preterm birth (PTB) is the leading cause of morbidity and mortality in infants <1 year of age. Intrauterine inflammation is a hallmark of preterm and term parturition; however, this alone cannot fully explain the pathobiology of PTB. For example, the cervix undergoes a prolonged series of biochemical and biomechanical events, including extracellular matrix (ECM) remodeling and mechanochemical changes, culminating in ripening. Vaginal progesterone (P_4_) prophylaxis demonstrates great promise in preventing PTB in women with a short cervix (<25 mm). We used a primary culture model of human cervical stromal fibroblasts to investigate gene expression signatures in cells treated with interleukin-1β (IL-1β) in the presence or absence of P_4_ following 17β-estradiol (17β-E_2_) priming for 7–10 days. Microarrays were used to measure global gene expression in cells treated with cytokine or P_4_ alone or in combination, followed by validation of select transcripts by semiquantitative polymerase chain reactions (qRT-PCR). Primary/precursor (MIR) and mature microRNAs (miR) were quantified by microarray and NanoString^®^ platforms, respectively, and validated by qRT-PCR. Differential gene expression was computed after data normalization followed by pathway analysis using Kyoto Encyclopedia Genes and Genomes (KEGG), Panther, Gene Ontology (GO), and Ingenuity Pathway Analysis (IPA) upstream regulator algorithm tools. Treatment of fibroblasts with IL-1β alone resulted in the differential expression of 1432 transcripts (protein coding and non-coding), while P_4_ alone led to the expression of only 43 transcripts compared to untreated controls. Cytokines, chemokines, and their cognate receptors and prostaglandin endoperoxide synthase-2 (PTGS-2) were among the most highly upregulated transcripts following either IL-1β or IL-1β + P_4_. Other prominent differentially expressed transcripts were those encoding ECM proteins, ECM-degrading enzymes, and enzymes involved in glycosaminoglycan (GAG) biosynthesis. We also detected differential expression of bradykinin receptor-1 and -2 transcripts, suggesting (prominent in tissue injury/remodeling) a role for the kallikrein–kinin system in cervical responses to cytokine and/or P_4_ challenge. Collectively, this global gene expression study provides a rich database to interrogate stromal fibroblasts in the setting of a proinflammatory and endocrine milieu that is relevant to cervical remodeling/ripening during preparation for parturition.

## Introduction

Cervical integrity is crucial for a successful human pregnancy. Throughout most of an uncomplicated gestation, the cervix provides a physical and immune barrier between the interior of the uterus and the vaginal microbiome (Word et al., 2007; Akgul et al., 2014; Vink and Feltovich, 2016). The cervix prepares for parturition by first slow and then rapid transition from an elongated, closed, and rigid structure to an orifice sufficiently soft and dilated to facilitate delivery of the fetus.

Human and animal studies have yielded a working model for the biomolecular underpinnings of cervical remodeling/ripening (Elovitz and Mrinalini, 2004; Word et al., 2007; Timmons et al., 2010; Yellon, 2017). Broadly speaking, normal remodeling is the product of changes in the organization and composition of the extracellular matrix (ECM) during pregnancy, including a decrease in cross-linked collagens I and III, changes in the composition of glycosaminoglycans (GAGs), especially elevated hyaluronan (HA) production, increased tissue hydration, and leukocyte infiltration (Sakamoto et al., 2005; Myers et al., 2009; Akgul et al., 2012; Dubicke et al., 2016).

Progesterone receptor (PR, NR3C3) signaling underpins many of the physiological processes that oppose untimely cervical dilation (Word et al., 2007). Two separate protein isoforms, PR-A (90 kDa) and PR-B (130 kDa), are encoded by a single gene differentially expressed via alternate promoter usage (Kastner et al., 1990; Stjernholm-Vladic et al., 2004). In rodents, pregnancy maintenance requires continued synthesis of progesterone (P_4_) by the corpus luteum. Systemic withdrawal of P_4_ during luteolysis in rodents evokes cervical ripening and labor at term, while premature cervical ripening is prompted by ovariectomy (Gonzalez et al., 2009). Additionally, pharmacologic antagonism of the PR by mifepristone (RU-486) promotes cervical ripening in animal models (Chwalisz et al., 1994). Conversely, administration of vaginal P_4_ reduces the incidence of preterm birth in women with a sonographically validated short cervix (Hassan et al., 2011).

There is strong evidence of the therapeutic utility of P_4_ for the prevention of untimely cervical ripening and preterm labor in at-risk women (Conde-Agudelo and Romero, 2016). However, unanswered questions persist regarding the mechanisms by which P_4_ influences the expression of inflammation-related genes, including cytokines and chemokines, extracellular matrix (ECM)-modifying enzymes, and bioactive lipid-generating enzymes (e.g., prostaglandin endoperoxidase synthase-2, PTGS-2; microsomal prostaglandin E synthase-1, mPGES; 5-lipoxygenase, 5-LOX) (Kniss, 1999; Sato et al., 2001; Ackerman et al., 2005) in gestational tissues, including the cervix. The overarching goal of the present study was to evaluate gene expression programs (including protein-coding and non-coding RNAs) executed in response to proinflammatory cytokine and/or PR:P_4_ stimulation in cervical fibroblasts. Our results revealed a multifaceted profile of gene expression in the cervical stroma, including (1) genes that are cytokine-responsive/P_4_-insensitive; (2) genes that are P_4_-sensitive/cytokine-independent; (3) genes that are cytokine-responsive and suppressed by P_4_; and (4) genes that are augmented by both cytokines and progesterone. These data provide a framework allowing us to construct gene networks involved in the manner by which PR signaling may prevent or delay inflammation-induced cervical ripening and consequent preterm labor. The ultimate goal of this work is that it allows us to identify and develop novel therapeutic targets for prevention and/or management of preterm labor, especially in at-risk women who manifest cervical insufficiency, a major cause of PTB.

## Materials and Methods

### Cervical Stromal Fibroblast Culture

With institutional review board approval (OSU Biomedical IRB Protocol Number 2013H0046), de-identified cervical tissues were obtained from premenopausal women undergoing hysterectomy for benign gynecological conditions. Primary human cervical stromal fibroblasts were isolated via outgrowth from explanted cervical stromal tissues as previously described (Ackerman et al., 2016b). For experiments, cells were grown to confluence in complete Dulbecco’s modified Eagle’s medium (DMEM, high-glucose, 4.5 g/l) supplemented with 10% fetal bovine serum (FBS), 50 μg/ml gentamicin sulfate, and 0.5 μg/ml amphotericin B (all from Invitrogen, Carlsbad, CA, United States). Next, the cells were rinsed with Dulbecco’s phosphate-buffered saline (DPBS) and then incubated in experimental medium containing phenol red-free DMEM/F12 (1:1) with 0.5% charcoal-stripped FBS (prepared in-house using charcoal-dextran extraction, clarification by centrifugation, and sterilization through a 0.2-μm filter) in the absence (0.001% ethanol vehicle control) or presence of 17β-estradiol (17β-E_2_, 10^–8^ M; Sigma-Aldrich, St. Louis, MO, United States) for 7–14 days to promote the expression of nuclear progesterone receptors (PRs) (Ackerman et al., 2016b). Finally, cells were incubated for 24 h in the absence or presence of P_4_ (10^–7^ M; Sigma-Aldrich), followed by challenge for 4 or 24 h with 0.2 ng/ml of human recombinant interleukin-1β (IL-1β; R&D Systems, Minneapolis, MN, United States) or an equivalent volume of vehicle (PBS with 0.1% bovine serum albumin, BSA). All experiments were performed between the 3rd and 7th passages after primary explant cultures were prepared. To validate the expression patterns of select transcripts following high-dimensional profiling studies, biological replicate experiments were performed using treatment conditions identical to those described above using a separate set of cell cultures. Cell cultures were routinely tested for mycoplasma species using an in-house PCR-based assay (MycoAlert^TM^, Lonza, Anaheim, CA, United States).

### RNA Extraction

Total RNA was extracted from harvested cells using TRIzol (Invitrogen). Following the addition of chloroform and centrifugation, the aqueous phase was mixed with an equal volume of 100% ethanol, applied to a miRNeasy spin column (Qiagen, Valencia, CA, United States) and processed according to the manufacturer’s protocol. The extraction procedure included on-column DNase I digestion using the RNase-Free DNase Set (Qiagen) to remove contaminating genomic DNA. RNA was quantified by absorbance at 260 and 280 nm using a NanoDrop 2000 spectrophotometer (Thermo Fisher, Hudson, NH, United States).

### Microarray Analysis

Total RNA (250 ng per sample) was processed using the Ambion WT expression kit (Austin, TX, United States) and labeled with Affymetrix GeneChip (Santa Clara, CA, United States) whole-transcript sense target labeling assay, followed by hybridization to the Affymetrix Human Transcriptome 2.0 array according to the manufacturer’s protocols. Following hybridization and scanning, quality control and robust multichip averaging were performed on the feature intensity files using the Affymetrix Expression Console software version 1.4. Gene-level differential expression analysis was subsequently performed using the Affymetrix Transcriptome Analysis Console software version 3.0 using the paired-sample analytical pipeline (one-way repeated-measure ANOVA), and the Benjamini–Hochberg false discovery rate (FDR)-controlling procedure (Reiner et al., 2003). For the default differential gene expression analysis, a linear fold-change threshold of ± 2 and an FDR of 10% was applied.

### Mature MicroRNA Profiling

Multiplexed mature miRNAs were profiled using the Human v3 miRNA Expression Assay (NanoString^®^ Technologies, Seattle, WA, United States). Total RNA (100 ng) was used as input for the nCounter^®^ miRNA sample preparation reactions according to the manufacturer’s instructions. Hybridization reactions were performed at 64°C for 18 h. Hybridized probes were analyzed using the nCounter digital analyzer. For each assay, a high-density scan (600 fields of view) was performed. The nSolver^®^ Analysis Software version 3.0 was used for technical normalization. Probes with low levels of expression (defined as less than the mean 2 ± SD of counts assigned to negative control probes, which was 33.04 in our data set) were omitted from subsequent analyses. Differential expression analysis of the NanoString^®^ data was performed using the edgeR (version 3.14.0) Bioconductor package (Robinson et al., 2010). The trimmed mean of *M*-value normalization was used together with the generalized linear model approach coupled with a paired-sample design matrix. Differential expression was determined using generalized linear model likelihood ratio tests. For FDR control, the Benjamini–Hochberg procedure was used (Reiner et al., 2003).

### Quantitative Real-Time Polymerase Chain Reaction (qRT-PCR)

To validate the expression of select mRNAs, 1 μg of total RNA was reverse transcribed to complementary DNA (cDNA) using oligo(dT)_12–18_ primers with SuperScript^®^ III Reverse Transcriptase (Life Technologies, Grand Island, NY, United States). Quantitative PCR was performed using an equal amount of cDNA per sample on a LightCycler 480 II System (Roche Applied Science, Indianapolis, IN, United States) using the following TaqMan^®^ primer/probe sets (Applied Biosystems, Foster City, CA, United States): *BDKRB1* (Hs00664201_s1), *BDKRB2* (Hs00176121_m1), *CXCL8* (Hs00174103_m1), *FKBP5* (Hs01561006_m1), *HAS2* (Hs00193435_m1), *HSD11B1* (Hs00194153_m1), *IL1B* (Hs01555413_m1), *IL6* (Hs00985639_m1), *IRAK3* (Hs00936103_m1), *MMP10* (Hs00233987_m1), *PTGES* (Hs00610420_m1), and *PTGS2* (Hs00153133_m1). The expression of *RPLP0* (4310879E) was used as a reference.

For primary/precursor miRNAs (pri-miRNAs), reverse transcription was performed using the high-capacity complementary deoxyribonucleic acid reverse transcription kit (Applied Biosystems), according to the manufacturer’s instructions. Each reaction comprised 10 μl of master mix (10 × reverse transcription buffer, deoxynucleotide triphosphates, reverse transcription random primers, MultiScribe^®^ reverse transcription enzyme (Thermo Fisher Scientific), ribonuclease inhibitor, and nuclease-free water) and 1 μg of RNA (in 10 μl reaction volume). For qRT-PCR, TaqMan^®^ gene expression master mix and TaqMan Pri-miRNA assays (Applied Biosystems) were used. The assays were Hs03303259_pri (*MIR146A*) and Hs03303349_pri (*MIR155*). For mature miRNAs, reverse transcription was performed with total RNA using the TaqMan microRNA reverse transcription kit (Applied Biosystems), per the manufacturer’s recommendations. Each reaction received 7 μl of TaqMan gene expression master mix, 5 μl of RNA (10 ng), and 3 μl of the reverse transcription primer appropriate for each target miRNA. TaqMan microRNA assays (Applied Biosystems) were used for qRT-PCR. The following assays were used: 000468 (hsa-miR-146a-5p) and 002623 (hsa-miR-155-5p). The relative abundance of each mRNA or miRNA was calculated by the comparative C_T_ method (Schmittgen and Livak, 2008).

### Bioinformatics

#### Pathway Analysis

Pathway analysis for microarray data was performed using the Gene Set Enrichment Analysis (GSEA) Java desktop application (version 2.2.2^1^) (Subramanian et al., 2005). For a given gene set, this algorithm calculates an enrichment score, which numerically reflects the degree to which a given dataset is overrepresented within a ranked list of gene expression data. The statistical significance of a given enrichment score is estimated using an empirical permutation test procedure, followed by correction for multiple-hypothesis testing. This analysis method tends to be more sensitive and robust than overrepresentation methods relying solely on differential expression with arbitrary cutoffs (Subramanian et al., 2005). Three databases of curated gene sets were obtained from an online repository^2^ (Merico et al., 2010) and queried: 1. Kyoto Encyclopedia of Genes and Genomes (KEGG)^3^; 2. Panther^4^; and 3. Gene Ontology (GO) molecular function^5^. All gene sets with 1–500 members were evaluated using 1000 gene-set permutations (as recommended when fewer than seven samples in any phenotype are available for analysis) by applying the default weighted enrichment statistic, the signal-to-noise ratio metric for ranking genes, and the default method for enrichment score normalization. Additionally, the Ingenuity Pathways Analysis (IPA) upstream regulator algorithm (Qiagen) was employed to infer upstream signaling events potentially contributing to observed gene expression signatures.

#### Transcription Factor Binding Motif Overrepresentation Analysis

The promoter regions (i.e., FASTA sequences ± 1000 bp relative to each transcription start site) of differentially expressed genes in select conditions were programmatically retrieved from the University of California Santa Cruz hg38 human genome assembly via the TogoWS SOAP API website^6^ (Katayama et al., 2010). The “multiple DNA sequences” algorithm of the Transcription Factor Affinity Prediction (Thomas-Chollier et al., 2011) (TRAP) Web Tools suite^7^ was used to probe these regulatory regions for transcription factor binding affinities based on position-specific scoring matrices present in the non-redundant JASPAR core vertebrate database (Mathelier et al., 2014) using a background model of all human promoter regions.

### Statistical Analysis

Statistical analyses were performed using the Kruskal–Wallis statistical test with *post hoc* testing using Dunn’s multiple comparison test when appropriate. A *p*-value < 0.05 was considered significant. Microarray and qRT-PCR experiments were replicated three times in duplicate (i.e., *n* = 6 samples per treatment). The data were tested for Gaussian distribution, and, if normally distributed, they were expressed as mean ± SEM and evaluated by analysis of variance (ANOVA) followed by *post hoc* testing by the method of Tukey using GraphPad Prism 8.0 software (San Diego, CA, United States).

## Results

### Common and Unique Features of IL-1β and Progesterone Genomic Responses

**FIGURE 1:**
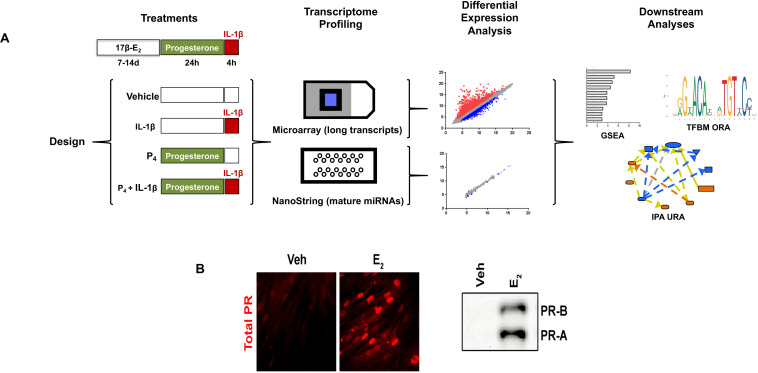
Experimental design and workflow. **(A)** Primary cultures of cervical fibroblasts were primed with 10^–8^ M 17β-estradiol (17β-E2) or 0.001% ethanol vehicle for 7–14 days in F12/DMEM++0.5% charcoal-stripped FBS. Cells were then treated with 10^–7^ M progesterone (P4) or vehicle for 24 h followed by a 4-h stimulation with 0.2 ng/ml interleukin-1β (IL-1*upbeta*) or vehicle (PBS/0.1% BSA). Total RNA was extracted to enrich for small RNAs and subjected to microarray analysis using the Affymetrix platform for long, coding transcripts and the NanoString^®^ platform for small, non-coding RNAs. After normalization, the data were subjected to differential expression analysis was conducted using the Affymetrix Transcription Analysis Console. Downstream relationships were evaluated using Gene Set Enrichment Analysis (GSEA), Transcription Factor Binding Motif (TFBM) analysis, and Ingenuity Pathway Analysis. The relative expression data are the mean and FDR *p*-value from three separate sets of biological replicates. **(B)** depicts the expression of PR-A and PR-B using immunofluorescence (left panel) or immunoblotting (right panel) following priming with 17β-E2.

**FIGURE 2:**
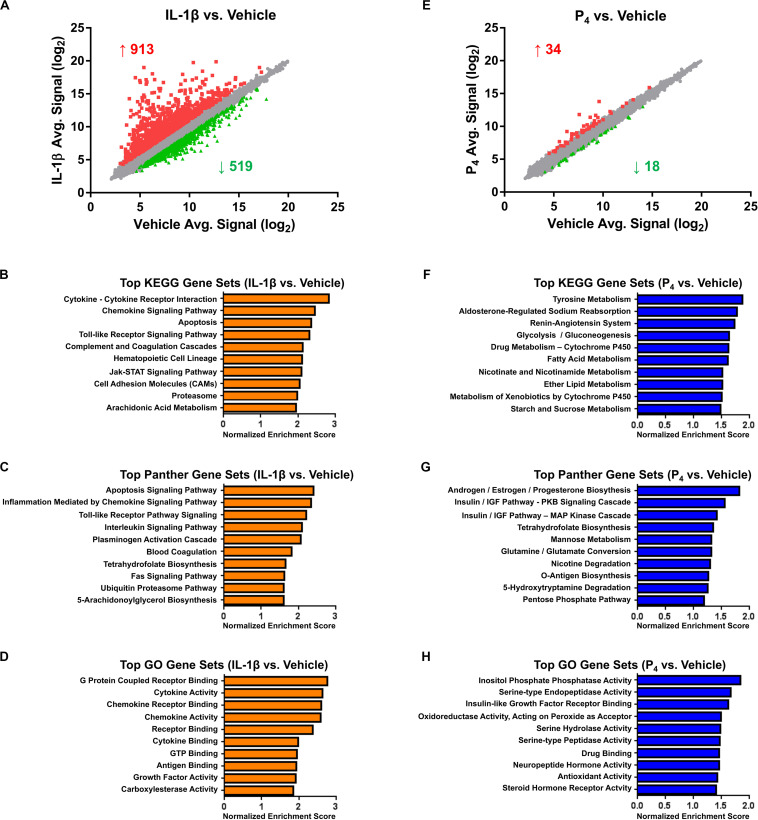
Microarray and bioinformatics analysis of differentially expressed genes in cervical stromal fibroblasts stimulated with IL-1β (0.2 ng/ml) or P_4_ (10^–7^ M) for hrs. Cells were primed with 17β-E2 (10^–8^ M) to induce progesterone receptors -A and -B and then challenged for 4 hrs with IL-1β **(A–D)** or P_4_ for 4 h (after a 24-h preincubation with the steroid) **(E–H)**, total RNA isolated and quantified and differential gene expression by microarray analysis. The normalized data are expressed as mean signal intensity (log_2_) and are from three separate experiments carried out in duplicate **(A,E)**. Bioinformatics analysis of differentially expressed transcripts using KEGG **(B,F)**, Panther **(C,G)** and GO **(D,H)** algorithms.

The experimental design for the studies described throughout this work is shown in Figure 1A. Importantly, cervical stromal fibroblasts were stimulated to elicit the expression of progesterone receptors (Figure 1B). After carrying out experiments, total RNA from each study group was extracted and purified, quantified, and subjected to microarray analysis of differential gene (coding and non-coding RNAs) expression. To confirm these results, we also conducted follow-up experiments using the identical design with biological replicates (i.e., a separate set of cell cultures not used for microarray analysis) to measure by qRT-PCR transcripts induced by either IL-1β, P_4_, or both agents incubated simultaneously. Extensive bioinformatics analysis was conducted using several publicly available genomic analytical tools (KEGG, Panther, and GO algorithms (Figure 2).

### IL-1β Induces a Broad Array of Transcripts

Cervical fibroblasts cultured as described (Ackerman et al., 2016b) were pretreated for 7–14 days with 10^–8^ M 17β-E_2_ to induce nuclear PR expression. Relative to vehicle-treated controls, microarray profiling of cells stimulated for 4 h with IL-1β (0.2 ng/ml) revealed changes in gene expression, with significant (minimum ≥two-fold change in expression and FDR <0.1) upregulation of 913 and downregulation of 519 transcripts (Figure 2A and Supplementary Table S1). As we observed previously in other intrauterine cell types, e.g., amnion mesenchymal fibroblasts (Li et al., 2011) and uterine decidual stromal cells (Ibrahim et al., 2016), highly IL-1β-induced transcripts included those encoding a wide array of cytokines and chemokines, enzymes involved in prostaglandin synthesis, bradykinin receptors-1 and -2, and matrix metalloproteinases, among others. Other prominent differentially expressed mRNAs included acute phase reactants, members of the complement family including tissue factor pathway inhibitor 2, and other proteases and their inhibitors (Supplementary Table S1). Intracellular signal transduction pathways differentially expressed following IL-1β treatment included the interferon (IFN), signal transducer and activator of transcription 5 (STAT5), and mitogen-activated protein kinase (MAP kinase)/phosphatase pathways (Supplementary Table S1).

Highly enriched pathways, as determined by the GSEA algorithms (KEGG, Panther, and GO, respectively), included those related to inflammatory signaling, apoptosis, blood coagulation, cell adhesion, and arachidonic acid metabolism (Figures 2B–D and Supplementary Table S2). Based on this gene expression signature, the IPA upstream regulator algorithm predicted activation of 65 and inhibition of 23 transcriptional regulators. As expected, following proinflammatory cytokine challenge, activation was inferred for nuclear factor-κB (NF-κB), components of the JAK-STAT signaling pathway, interferon regulatory factors (IRFs), activator protein-1 (AP-1) subunits, and Forkhead box (FOX) transcription factors, among many others (Supplementary Table S3). Confirming these predictions, a complementary promoter scanning analysis (TRAP analysis) of the differentially regulated genes revealed overrepresentation of canonical binding motifs corresponding to several of these transcription factors, including NF-κB (i.e., the NF-kappaB, NFKB1, REL, and RELA model matrices), FOX (i.e., the Foxq1, FOXF2, FOXO3, FOXD1, Foxa2, and FOXI1 matrices), IRF (i.e., the IRF1 and IRF2 matrices), STAT (i.e., the STAT1 and STAT3 matrices), and AP1 (Supplementary Table S4).

Although not prominently represented in the pathway analyses, given the importance of the ECM in cervical remodeling, we surveyed IL-1β-responsive transcripts for changes in genes responsible for ECM integrity, including proteoglycans, fibrous proteins, and genes associated with ECM biosynthesis and degradation. IL-1β induced the expression of three proteoglycan-related proteins (hyaluronan receptor*/CD44*, syndecan-4*/SDC4*, and serglycin/*SRGN*) and attenuated the expression of an additional two (decorin/*DCN* and structural maintenance of chromosome 3/chondroitin sulfate proteoglycan-6/*SMC3*) proteins. IL-1β also induced the expression of elastin (*ELN*), a basement membrane-associated procollagen (collagen type IV, α1 chain/*COL4A1*), and several genes involved in glycosaminoglycan (GAG) biosynthesis (β-1,4-galactosyltransferase-1/*B4GALT1*, carbohydrate sulfotransferase-11/*CHST11*, exostosin glycosyltransferase-/*EXT1*, fucosyltransferase-8/*FUT8*, hyaluronan synthase-2/*HAS2*, heparan sulfate-glucosamine 3-sulfotransferase-3B1/*HS3ST3B1*, and/ST3 β-galactosidase α-2,3-sialytransferase 1/*3GAL1*), while decreasing the expression of collagen type III, α1 chain/*COL3A1*, a major fibrillar procollagen in the cervical ECM Supplementary Table S1.

### Progesterone Regulates a Modest Set of Genes

In contrast to the surfeit of differentially expressed genes observed following IL-1β stimulation, the transcriptional response to progesterone (10^–7^ M, a dose similar to the maternal circulating levels late in human gestation) (Word et al., 2007) was modest. Compared to cells receiving vehicle alone, cervical fibroblasts incubated with P_4_ exhibited expression changes in only 52 genes, 34 of which were upregulated and 18 downregulated (Figure 2E and Supplementary Table S5).

Top-ranking pathways (analyzed by KEGG, Panther, and GO algorithms) associated with this gene expression signature included those related to the amino acid metabolism, xenobiotics, and fatty acid biosynthesis and catabolism, in addition to cortisol biosynthesis, insulin-like growth factor (IGF) signaling, and sodium reabsorption and the renin–angiotensin system (RAS) (Figures 2F–H and Supplementary Table S6). Based on this expression pattern, the IPA upstream regulator algorithm correctly predicted activation of the PR and inferred inhibition for a small number of inflammatory and growth regulators (Supplementary Table S7). Promoter scanning analysis revealed overrepresentation of the NR3C1 model matrix corresponding to the consensus binding motif of the glucocorticoid receptor (GRE: 5’RGRACANNNTGTYC3’, where R = purine, Y = pyrimidine, N = any nucleic acid) (Supplementary Table S8). This was somewhat expected, inasmuch as the response elements for the glucocorticoid and progesterone receptors are quite similar (Lieberman et al., 1993; Nelson et al., 1999), and a separate PR response element model was not included in the non-redundant JASPAR vertebrate matrix database used for this query.

Unexpectedly, however, these promoter regions were also enriched for NF-κB-binding motifs. Given that we expected NF-κB-responsive genes to be induced following cytokine challenge, we then compared the extent of overlap between the genes differentially expressed following IL-1β and P_4_ co-stimulation. We found that a considerable proportion (31%) of the genes influenced by P_4_ were also IL-1β responsive; specifically, nine genes were upregulated in both conditions. In addition, eight genes were downregulated following either treatment. Interestingly, most genes induced by either treatment were found to have roles in mitigating inflammation based on literature review (Table 1).

**TABLE 1:** Characteristics of transcripts induced by both P4 and IL-1β based on microarray profiling.

Gene symbol	Description	IL-1β vs. vehicle fold change	P_4_ vs. vehicle fold change	P_4_ + IL-1β vs. vehicle fold change	Notes (references)
ALDH1A3	aldehyde dehydrogenase 1 family, member A3	2.25	2.57	4.43	Androgen-responsive gene in human prostate cancer epithelial cells (Trasino et al., 2007); participates in retinoic acid production and the metabolism of acetaldehyde, some amino acids, lipid peroxidation products, and exogenous chemicals (Duan et al., 2016).
CRISPLD2	cysteine-rich secretory protein LCCL domain containing 2	7.12	3.56	10.7	Progesterone receptor target gene in uterine cells (Yoo et al., 2014); blocks HMGB1-induced inflammation (Zhang et al., 2016); prevents lipopolysaccharide binding to target cells (Wang et al., 2009); responsive to both glucocorticoids and IL-1β in airway smooth muscle cells and negatively regulates pro-inflammatory cytokine function (Himes et al., 2014)
FKBP5	FK506 binding protein 5	2.18	4.13	4.2	Induced by glucocorticoids and progestins via hormone response elements (Hubler and Scammell, 2004); attenuates progestin responsiveness in cell models (Hubler et al., 2003).
HSD11B1	hydroxysteroid (11-beta) dehydrogenase 1	30.41	2.57	93.12	Reversibly reduces inactive cortisone to the active glucocorticoid receptor agonist cortisol (Oppermann et al., 1997); induced by progesterone in human endometrial stromal cells (Kuroda et al., 2013); responsive to IL-1α in human ovarian surface epithelium (Yong et al., 2002).
IRAK3	interleukin-1 receptor-associated kinase 3	6.77	2.5	19.75	Negative regulator of Toll/interleukin receptor signal transduction (Kobayashi et al., 2002); prevents neutrophil-dependent lung injury during influenza infection of the respiratory tract (Seki et al., 2010)
MMD	monocyte to macrophage differentiation-associated	5.42	2.48	9.64	Up-regulated during monocyte differentiation (Rehli et al., 1995); induced by lipopolysaccharide in macrophages (Liu et al., 2012); enhances cytokine and nitric oxide production in macrophages (Liu et al., 2012); plays a critical role in heart morphogenesis (Huang et al., 2012); inhibits growth of lung cancer cells (Li and He, 2014).
PLPP3	phospholipid phosphatase 3	4.16	2.93	4.86	Promotes anti-inflammatory phenotype and maintains vascular integrity of endothelial cells (Wu et al., 2015); promotes endothelial barrier function and limits sensitivity to inflammation-induced vascular leaks (Panchatcharam et al., 2014)
PPP4R4	protein phosphatase 4, regulatory subunit 4	4.39	3.47	8.18	Regulatory subunit for phosphoprotein phosphatase 4 PPP4C, modulates the latter’s role in microtubule dynamics, DNA damage checkpoint recovery, apoptosis, and tumor necrosis factor alpha signaling (Chen et al., 2008); induced by preovulatory LH surge in ovarian steroidogenic cells (Christenson et al., 2013).
SLC39A8	solute carrier family 39 (zinc transporter), member 8	36.2	2.28	47.64	Functions in the cellular import of zinc at the onset of inflammation (Begum et al., 2002); NF-κB target gene that suppresses inflammatory signaling in zinc-dependent fashion (Liu et al., 2013).

### Progesterone Selectively Modulates Global Cytokine-Elicited Transcription

**FIGURE 3:**
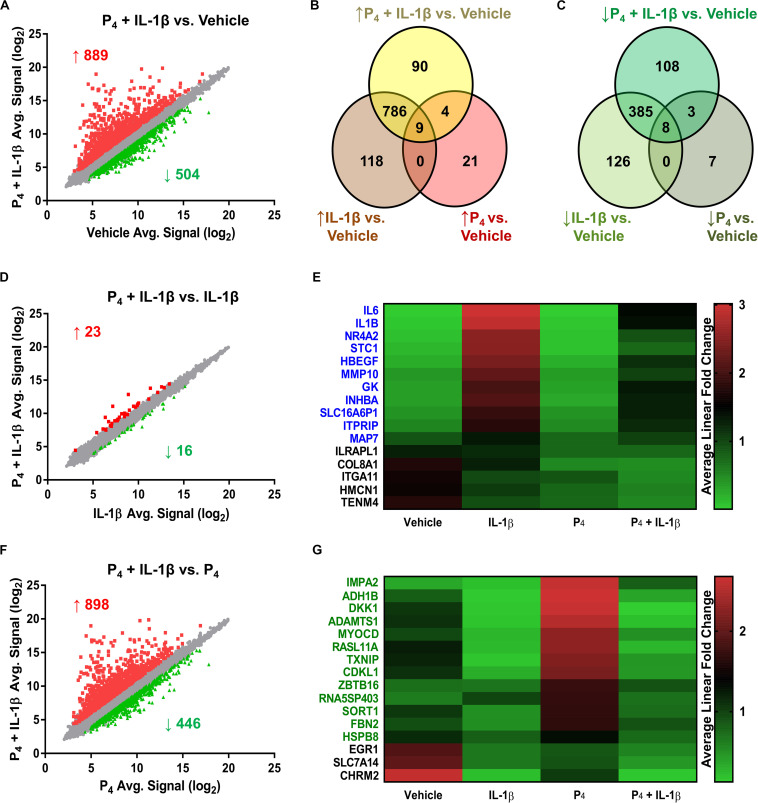
Differential gene expression in cervical stromal fibroblasts stimulated with either IL-1β (0.2 ng/ml), P_4_ (10^–7^ M) or both for 4 h followed by microarray analysis and data normalization. **(A)** shows the scatter plot and Venn diagrams comparing upregulated **(B)** and downregulated transcripts **(C)** in response to IL-1β +P_4_ or vehicle. **(D)** Scatter plot and **(E)** heat map of differentially expressed genes in response to IL-1β or IL-1β + P_4_. **(F)** Scatter plot and **(G)** heat map of differentially expressed genes in response to P_4_ or IL-1β +P_4_.

Relative to control cells, the global cellular response to IL-1β + P_4_, like that following IL-1β exposure alone, was considerable: 889 genes exhibited induced expression, while 504 showed diminished expression (Figures 3A–C and Supplementary Table S9). The Jaccard similarity indices between these treatment groups were 0.79 and 0.62 for upregulated and downregulated genes, respectively. A minority (24 transcripts) of the genes differentially expressed by IL-1β + P_4_ were also regulated by P_4_ alone. Among the 17 genes responsive to both IL-1β and P_4_ mentioned previously were ABI family member 3 binding protein/*ABI3BP*, collagen type VIII α1 chain/*COL8A1*, estrogen receptor 1/*ESR1*, osteomodulin/*OMD*, plexin domain containing 2/*PLXDC2*, prolactin receptor/*PRLR*, and secreted protein acidic and cysteine-rich-like 1/*SPARCL1*.

To assess the degree to which co-incubation with P_4_ modified global IL-1β-inducible gene expression, we next compared the IL-1β and IL-1β + P_4_ treatment groups. Of the 39 genes differentiating these two groups, 16 were downregulated by combined IL-1β + P_4_ treatment relative to IL-1β alone (Figure 3B and Supplementary Table S10). Of these, 11 genes were responsive when cells were incubated with IL-1β alone, including two proinflammatory interleukins (*IL1B*, *IL6*), a matrix metalloproteinase (*MMP10*), and the inhibin beta A subunit (*INHBA*) (Figure 3E). Finally, to determine how cytokine stimulation affected P_4_-induced gene expression, we compared the P_4_ and IL-1β + P_4_ treatment groups (Figure 3F and Supplementary Table S11). Among the 446 genes downregulated by IL-1β under these conditions were 16 genes differentially regulated by P_4_ alone. Among the 13 progestin-responsive genes exhibiting diminished expression when incubated in the presence of IL-1β were those encoding the transcription factor myocardin (*MYOCD*), alcohol dehydrogenase 1B beta (*ADH1B*), the WNT signaling pathway inhibitor Dickkopf Inhibitor 1 (*DKK1*), and the ECM scaffold glycoprotein fibrillin 2 (*FBN2*) (Figure 3G).

**FIGURE 4:**
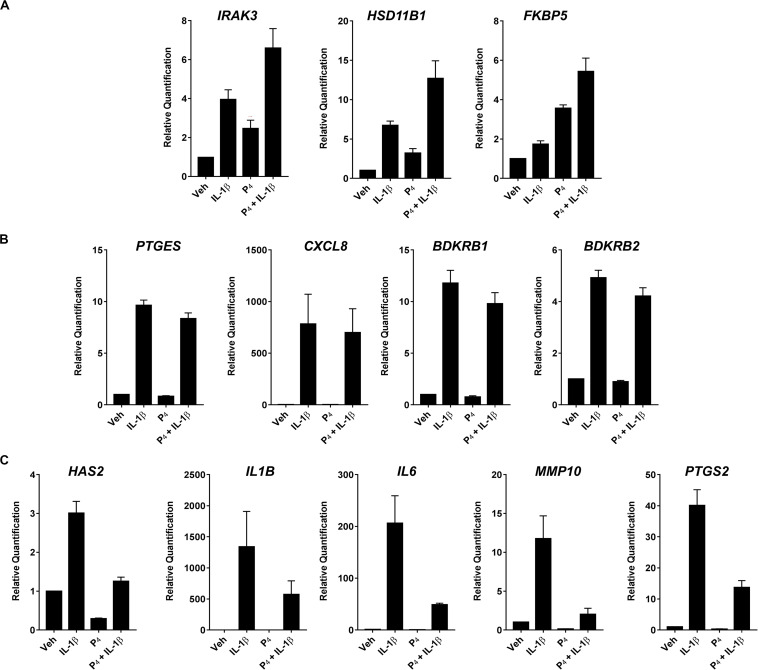
Quantitative RT-PCR of select differentially expressed transcripts in cervical stromal fibroblasts stimulated with vehicle, IL-1β, P_4_ or IL-1β+P_4_ for 4 h. **(A)** Additive or synergistic relationship between P4 and IL-1 **(B)** No effect of P4 **(C)** Inhibitory effect of P4. After treatments, total RNA was isolated and quantified and primer sets were used to analyze individual transcripts in three separate experiments carried out in duplicate. Data are expressed as mean ± SEM of relative signal intensity (target/human acidic ribosomal protein).

To validate the initial profiling results, we performed qRT-PCR for select transcripts in experiments using separate biological replicates. These transcripts were categorized into three general expression patterns: (1) additive/synergistic expression in response to IL-1β + P_4_ compared to either treatment alone (e.g., *IRAK3*, *HSD11B1*, and *FKBP5*) (Figure 4A); (2) induction by IL-1β with no significant response to P_4_ in the absence or presence of IL-1β (e.g., *BDKRB1*, *BDKRB2*, *PTGES*, and *CXCL2*) (Figure 4B); and (3) IL-1β-inducible expression attenuated when co-incubated with P_4_ (e.g., *HAS2*, *IL1B*, *IL6*, *MMP10*, and *PTGS2*) (Figure 4C).

### Non-coding RNAs Are Induced by Cytokine and Progestin

The microarray chip analysis included both coding and non-coding RNA transcripts. We detected several miRNAs (host genes, pri-/pre-miRNAs, and mature miRNAs) and long, intergenic non-coding (LINC) RNAs in our microarray studies that were selectively upregulated by IL-1β stimulation. In addition, 12 non-coding RNAs, including five LINC RNAs, were downregulated when cells were challenged with the cytokine (Supplementary Table S1). In subsequent experiments, using biological replicates (samples separate from those used in the microarray studies), we used NanoString and qRT-PCR to further assess non-coding RNA expression. Given the modest differential expression of LINC RNAs in our experiments, we did not pursue these transcripts in follow-up analyses.

**FIGURE 5:**
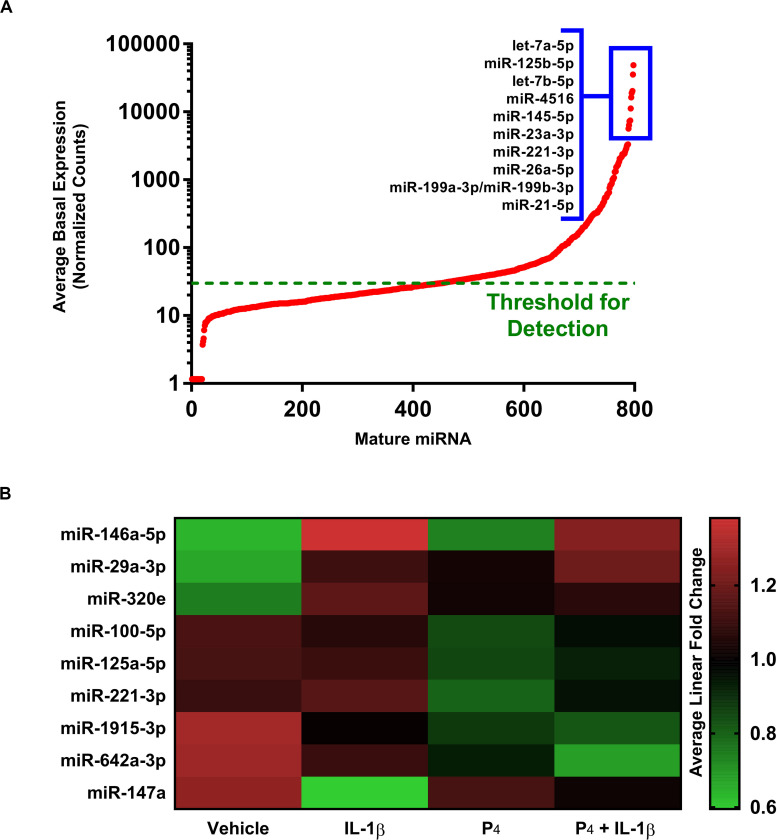
Differential expression of microRNAs in cervical stromal fibroblasts stimulated with IL-1β, P_4_ or co-incubated with IL-1β + P_4_. **(A)** Basal (normalized counts) levels of the most prominently expressed mature miRNAs above the detection limit in cervical stromal cells using the unamplified NanoString^®^ target counting platform. **(B)** Heat map of differentially expressed mature miRNAs following treatment with vehicle, IL-1β, P_4_ or co-incubated with IL-1β + P_4_. Data are from three separate experiments carried out in duplicate.

We surveyed the expression of 800 mature miRNAs simultaneously using the non-amplification-based NanoString platform. Overall, the expression of individual miRNAs spanned five orders of magnitude, with 346 transcripts having average normalized counts above the threshold for detection (Figure 5A). The most highly expressed mature miRNAs under basal (vehicle treatment only) conditions were let-7a-5p, let-7b-5p, miR-125b-5p, miR-145-5p, and miR-4516. When subjected to differential gene expression analysis, nine mature miRNAs were differentially expressed under any treatment condition (Figure 5B and Supplementary Table S12), with expression changes on the order of two-fold or smaller. This was in striking contrast to the rather large fold changes in expression estimated at the level of miRNA host genes (i.e., immature pri-/pre-miRNA transcripts). For example, our microarray data indicated that the host gene for miR-155 changed by 20-fold in response to IL-1β (Supplementary Table S1), yet the corresponding mature miR-155-5p exhibited no discernible change in expression in the NanoString dataset (not shown). Using qRT-PCR, we found that the pri-/pre-miRNAs *MIR146A* and *MIR155* exhibited large changes in expression (166- and 32-fold, respectively) in response to IL-1β, yet the corresponding mature species changed only modestly (miR-146a-5p and miR-155-5p) (Figure 5).

In our studies of miRNA expression in response to IL-1β and/or P_4_ exposure, we used the microarray platform to measure pri-/pre-miRNA transcripts while NanoString profiling was used to evaluate mature, fully processed miRNA species. We were surprised to learn that, while many pri-/pre-non-coding RNA host genes (including long non-coding and coding transcripts) were differentially expressed in cytokine-stimulated cells, far fewer full-processed miRNAs were observed (Supplementary Table S1 and Figure 5). This led us to postulate that this was due, at least in part, to the fact that while microarray analysis requires amplification of target transcripts, NanoString does not utilize a pre-amplification step prior to measurements.

**FIGURE 6:**
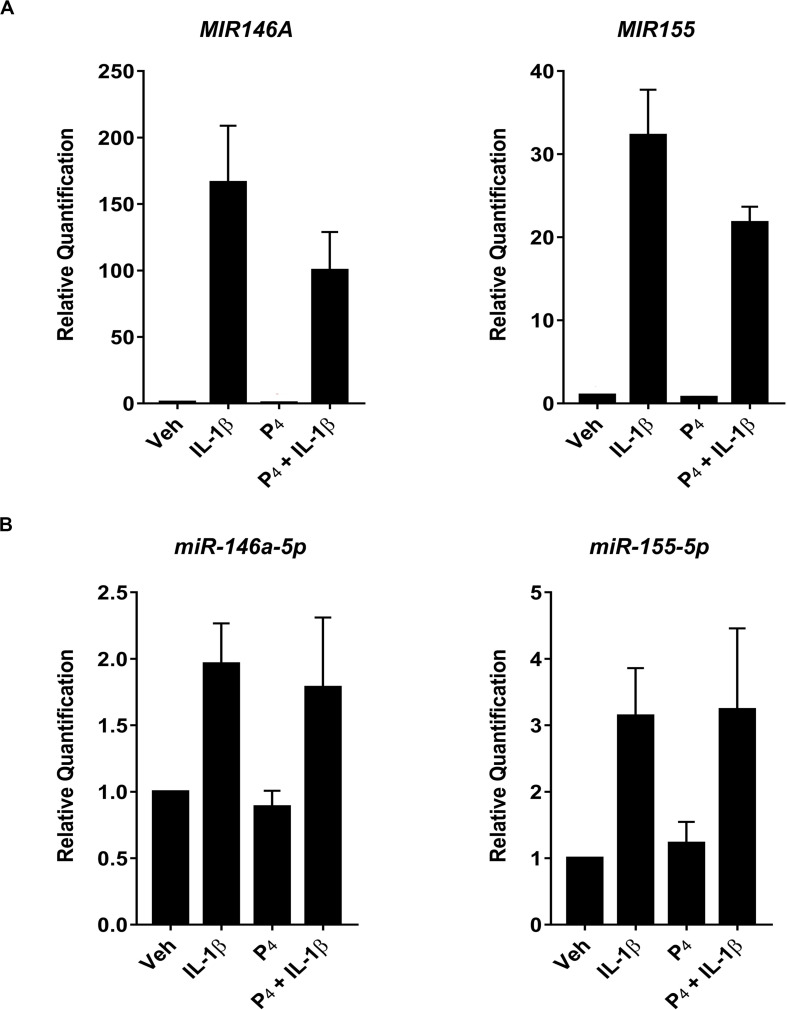
Quantitative RT-PCR measurement of select primary/precursor and mature miRNA transcripts in cervical stromal fibroblasts from vehicle-, IL-1β-, P_4_, or IL-1β + P_4_-treated cells. Three separate experiments (carried out in duplicate) using biological replicates were incubated with the test agents listed above for 4 h, and then total RNA was isolated, quantified and subjected to RT-PCR using primers specific for either the primary/precursor or mature miRNA transcripts. **(A)** Primary/precursor miRNA transcripts. **(B)** Fully processed, mature miRNAs. Data are expressed as mean ± SEM of relative intensity (target/human acidic ribosomal protein) and were analyzed by one-way ANOVA followed by Tukey’s test for differences (*p* < 0.05, considered significant).

To test this possibility, we compared directly two highly expressed miRNA transcripts (miR-146a and miR-155) using qRT-PCR of both immature and mature miRNAs. Figure 6A demonstrated that IL-1β stimulated robust upregulation of primary/precursors, MIR146a and MIR155, while co-incubation of IL-1β-treated cells with P_4_ attenuated MIR146a and MIR155 expression. In contrast, when we used the same analytical platform (qRT-PCR) to evaluate mature miRNAs, we found that, while IL-1β induced miR-146a-5p and miR-155-5p, P_4_ had no inhibitory effect on these two non-coding transcripts (Figure 6B).

## Discussion

### Principal Findings

The current work investigated for the first time the modulation of cytokine-induced gene expression by P_4_ using a simple, clinically relevant *in vitro* model of human cervical stromal fibroblasts. We chose to study the stroma based on the fact that the cervical stroma is the primary tissue that undergoes biochemical and biophysical remodeling allowing the fetus to descend the birth canal at the time of delivery (Malmstrom et al., 2007; Dubicke et al., 2016; Yellon, 2017; Shukla et al., 2018). Using a microarray platform and qRT-PCR-based validation, we demonstrated that IL-1β stimulation of stromal cells elicited changes in a panoply of genes encoding proinflammatory mediators, including cytokines and chemokines, arachidonic acid synthesizing enzymes, ECM-synthesizing enzymes, MMPs, and other proteases, and intracellular signaling proteins and transcription factors.

We also examined the regulation of genes in response to incubation with physiological levels of progesterone that are present at the end of pregnancy. In contrast to previous reports by DeMayo and colleagues (Jeong et al., 2005; Rubel et al., 2012), we detected relatively few genes that were directly controlled by P_4_. With the exception of a few genes which were upregulated >four-fold (i.e., the matricellular protein SPARC-like 1/hevin, 18.66-fold; osteomodulin, 7.3-fold; inositol monophosphatase 2, 6.39-fold; alcohol dehydrogenase 1B, 5.36-fold; FK506-binding protein 5, 4.13-fold), most progestin-regulated genes were only modestly upregulated or downregulated in stromal cells.

### Three Patterns of Progesterone Regulation of Cytokine-Driven Gene Expression

In contrast to gene expression in the context of progestin alone, co-incubation of cervical stromal cells with IL-1β and P_4_ elicited three distinct patterns of regulation. One cohort of transcripts was profoundly upregulated by treatment with IL-1β but was unaffected by P_4_ treatment alone (Figure 4B). A second set of transcripts exhibited IL-1β-stimulated upregulation that was almost completely suppressed by co-incubation with P_4_ (Figure 4C). Notably, this set of genes was represented by proinflammatory cytokines (IL-1β and IL-6), the enzyme responsible for hyaluronan synthesis (i.e., HAS-2) (Garantziotis and Savani, 2019) and the rate-limiting enzyme in proinflammatory prostaglandin biosynthesis (i.e., PTGS-2) (Kniss, 1999). This can be interpreted as the subset of genes for which progestins exert anti-inflammatory actions.

Interestingly, there was a third set of IL-1β upregulated transcripts that was further elevated either additively or synergistically when cervical stromal cells were co-incubated with cytokine and progestin, including the steroid hormone receptor chaperone FK506-binding protein 5 (FKBP5) (Storer et al., 2011) and 11β-HSD1, the enzyme that converts biologically inert cortisone into bioactive cortisol (Tomlinson et al., 2004; Chapman K. et al., 2013; Figure 4A). Thus, the previously undescribed finding of three different patterns of gene expression exposed to cytokine and progestin in combination suggests that P_4_ exerts complex regulatory control in the cervical stromal compartment and that, strictly speaking, it is not simply an anti-inflammatory steroid hormone. The suppression of proinflammatory genes induced by IL-1β by physiological concentrations of P_4_ was predicted from previous studies in decidual stromal cells (Cakmak et al., 2005) and myometrial cells (Mendelson, 2009; Shynlova et al., 2013; Georgiou et al., 2016; Amini et al., 2019).

### Regulation of Non-coding RNAs by Cytokine and Progestin

Analysis of the global expression of non-coding RNAs in cervical stromal cells treated with cytokine in the presence or absence of progestin revealed that miRNAs represent a relative minority of regulatory inputs to gene expression. Upregulated non-coding transcripts measured by microarray included both miRNAs encoded within host genes and LINC RNAs. The most highly upregulated miRNA transcript in IL-1β-treated cells was miR-155. MicroRNA-155 (hsa-mir155, coded within B-cell integration cluster (BIC) of non-coding transcripts located on chromosome 21, TargetScan, Release 7.1, June 2106; miRbase, Release 22.1, October 2018) (Madden et al., 2010; Kozomara and Griffiths-Jones, 2014) has been reported by many investigators to be induced in the setting of inflammation (O’Connell et al., 2007; Tili et al., 2007; Ceppi et al., 2009; Quinn and O’Neill, 2011; Xu et al., 2013) as a means to control tissue damage. We have recently demonstrated in decidual stromal cells that IL-1β causes the rapid and sustained upregulation of miR-155 (Ibrahim et al., 2016), and this was further confirmed using tissues isolated from patients who had a preterm delivery (Ackerman et al., 2016a). Thus, cytokine induction of miR-155 can be interpreted as a potential feedback loop to prevent tissue injury in the face of unrestrained acute inflammation (Baltimore et al., 2008; Contreras and Rao, 2012). One proposed mechanism by which miR-155 thwarts unwanted inflammation is by inhibition of the canonical inflammatory transcription factor NF-κB (Ma et al., 2011; Boldin and Baltimore, 2012).

Interestingly, in the NanoString platform, which does not amplify transcripts prior to their measurement, we were unable to detect significant differential expression of miR-155 following IL-1β stimulation, suggesting that, although induced by cytokine treatment the absolute abundance of this miRNA is quite low. When we analyzed miRNA expression using amplification-based qRT-PCR, we noted that, while the primary/precursor transcripts for miR-146a and miR-155 were substantially upregulated by IL-1β incubation, there was no significant induction of the corresponding mature miRNAs. These data further indicate that the regulation of miRNAs and their modulation of cytokine-induced gene expression can be quite modest (O’Neill et al., 2011; Ibrahim et al., 2016).

One of the most significantly downregulated miRNAs in IL-1β-treated cervical stromal cells was miR-143. This regulatory RNA transcript has been shown to target the COX-2 mRNA, suggesting that expression of this RNA in the basal state may prevent COX-2-mediated inflammatory prostaglandin biosynthesis (Kim et al., 2011; Pham et al., 2013).

### Upregulation of BDKRs and HAS-2 Contributes to Cervical Remodeling

Among the transcripts that were upregulated by cytokine but unaffected by progestin were the bradykinin receptors (BDKRB1 and BDKRB2). Bradykinin is a nonapeptide (H_2_N–Arg–Pro–Pro–Gly–Phe–Ser–Pro–Phe–Arg–COOH) member of the kallikrein–kinin system (Burch et al., 1989) that is generated following the activation of plasma or tissue kallikrein and subsequent cleavage of kininogen into several smaller products (Moreau et al., 2005; Bryant and Shariat-Madar, 2009; Bjorkqvist et al., 2013). Bradykinin is implicated in several pathophysiological events, including coagulation (Del Rosso et al., 2011; Wu, 2015; Weidmann et al., 2017) and thrombosis, fibrinolysis (Del Rosso et al., 2008), and acute inflammation (Kaplan and Joseph, 2014; Wu, 2015; Schmaier, 2016). Among the physiological functions of bradykinin is vascular permeability (Bjorkqvist et al., 2013; Couture et al., 2014) and smooth-muscle contractility (Ricciardolo et al., 2018). It is possible that bradykinin, acting via BDKR-B1 and/or -B2, mediates increased tissue hydration in the cervix during ripening by altering endothelial cell junctions (Couture et al., 2014). HAS-2, the enzyme that catalyzes the synthesis of hyaluronan (hydrophilic GAG) that accumulates in the stroma during ripening, is upregulated by IL-1β, and the induction is attenuated by P_4_. Thus, HAS-2- and BDKR-mediated functions of bradykinin may act in concert to orchestrate the softening that occurs during cervical ripening in preparation for parturition. To our knowledge, the current work is the first report of cytokine-induced bradykinin receptor expression in the cervical stroma. While P_4_ had no effect on IL-1β induction of BDKR-B1 or -B2, HAS-2 transcripts were nearly abolished when IL-1β-treated cells were co-incubated with P_4_. This observation provides a means by which progestins may prevent untimely hyaluronan synthesis and enhanced tissue hydration in the cervical stroma.

### ECM Proteins and ECM-Modifying Enzymes

The regulation of ECM composition is a major determinant of the biomechanical features of cervical ripening in preparation for the onset of labor (Myers et al., 2008, 2009; House et al., 2009). During the initial phases of cervical remodeling in early pregnancy, collagens type I and III are highly organized and cross-linked via lysyl oxidase and lysyl hydroxylase (Hassan et al., 2009; Akins et al., 2011). This provides a rigid and closed cervix that resists the gravitational and contractile forces that would otherwise contribute to opening of the cervical os and premature delivery of the fetus (Danforth, 1983; Hao et al., 2018). Later in pregnancy, cross-linked collagens are replaced by randomly oriented fibers, increased hyaluronan accumulation, and tissue hydration (Garantziotis and Savani, 2019). Our *in vitro* studies demonstrated that treatment of cells with IL-1β downregulated collagen I and III and lysyl oxidase mRNA expression (Supplementary Table S1). Moreover, cytokine exposure of cervical stromal cells led to upregulated HAS-2 expression that was antagonized by simultaneous incubation with P_4_. These results are consistent with previous findings by Elovitz’s group that progestational agents act to maintain cervical integrity in a murine model of parturition (Xu et al., 2008).

We also showed that IL-1β caused the upregulation of several MMPs (i.e., MMP1/interstitial collagenase, 3/stromelysin 1, MMP10/stromelysin 2, and MMP12/macrophage elastase) by ≥five-fold. These data are consistent with previous reports using human cervical fibroblasts (Yoshida et al., 2002). Combined incubation of stromal cells with IL-1β and P_4_ resulted in ∼ 60% reduction in MMP10 mRNA expression (see Figure 4 and Supplementary Tables S1, S9). In contrast, co-incubation of cells with cytokine and progestin led to less pronounced diminution in MMP1 (∼15%) and MMP3 (∼42%) and no effect on MMP12 expression. These data suggest that P_4_ has minimal effects on the key proteinases involved in tissue remodeling in the cervix.

We also detected the upregulation of several GAG synthases (i.e., B4GALT1, CHST11, EXT1, 3GAL1, FUT8, HS3STB1, and ST3) by IL-1β which was largely unaffected by P_4_. Importantly, however, IL-1β-induced HAS-2 mRNA expression was strongly inhibited by P_4_ co-incubation (see Figure 4), consistent with a previous report by Uchiyama et al. (2005). Taken together, these data indicate that, while inflammatory cytokines have wide-ranging effects on molecules involved in ECM biosynthesis and turnover, progestin treatment has a modest effect on these functions.

### Synergistic Induction of 11β-HSD1 by Cytokine and Progestin

The expression of 11β-HSD1, the enzyme that converts biologically inert cortisone into bioactive cortisol in several tissues, was induced several folds in stromal cells incubated with IL-1β. In addition, P_4_ treatment alone led to a 3-5-fold increase in 11β-HSD1 mRNA abundance, while 11β-HSD2, the enzyme that inactivates cortisol to cortisone, was unaffected by either cytokine or progestin exposure (data not shown) (Seckl, 2004). Surprisingly, when we combined exposure to IL-1β and P_4_ in cervical stromal cells, we detected a very robust synergistic upregulation of the 11β-HSD1 gene and no change in 11β-HSD2 gene expression. Previous studies have reported that proinflammatory cytokines can induce 11β-HSD1 leading to local synthesis of cortisol (Chapman K. et al., 2013). Chapman and Seckl have suggested that local expression of 11β-HSD1 and conversion of cortisone to cortisol provides a means to dampen acute inflammation that could lead to chronic tissue injury if left unchecked (Seckl, 2004; Chapman et al., 2006; Chapman K.E. et al., 2013). Ahasan et al. (2012) reported that IL-1β caused upregulation of 11β-HSD1 as an anti-inflammatory response in mesenchymal stromal cells. Similarly, Hardy et al. (2008, 2016) demonstrated upregulation of 11β-HSD1 mRNA expression in acute inflammation in skeletal muscle and in the context of inflamed synovial tissue with patients with rheumatoid arthritis (Hardy et al., 2008).

## Conclusion and Future Directions

The current work has demonstrated that incubation of cervical stromal fibroblasts with a proinflammatory cytokine leads to a robust global gene expression profile that includes predicted inflammatory and anti-inflammatory transcripts (e.g., cytokines, chemokines, enzymes involved in bioactive lipid synthesis, signaling proteins, and transcription factors). In addition, we also demonstrated several other groups of genes expressed in response to IL-1β, including ECM proteins and ECM-modifying enzymes (e.g., matricellular proteins, MMPs, other proteases, and glycosyltransferases driving the biosynthesis of GAGs). Finally, several non-coding RNAs were identified, including miRNAs and LINCRNAs.

A major objective of the study was to examine the role played by progesterone in governing cytokine-driven gene expression. In this regard, we were somewhat surprised to observe that a relatively modest cohort of stromal cell transcripts was regulated either positively or negatively by progestin. P_4_ was found to inhibit only a subset of stromal cell genes following IL-1β treatment, suggesting that this hormone exerts only partial anti-inflammatory activity, at least in the cervical stroma. For example, while P_4_ completely abolished cytokine-mediated IL-6 mRNA expression, it was ineffective at inhibiting CXCL8/IL-8 gene expression.

Finally, this work reported for the first time that IL-1β and P_4_ conspired to upregulate a few genes, e.g., FKBP5, a steroid receptor chaperone and 11β-HSD1, the enzyme that converts inert cortisone into bioactive cortisol. In addition, we discovered for the first time that IL-1β upregulated the expression of bradykinin receptors B1 and B2 that mediate the actions of kallikrein products in vascular permeability and other events in the inflammatory cascade. These latter two observations open a new avenue for future investigations into the interaction of cytokines and progestins in governing the events leading to cervical ripening in preparation for parturition.

## Data Availability Statement

The microarray data is available at GEO (GSE154571).

## Ethics Statement

The studies involving human participants were reviewed and approved by The Ohio State University Biomedical Institutional Review Board. The patients/participants provided their written informed consent to participate in this study.

## Author Contributions

DK conceived of the study, designed the experiments, analyzed the data, wrote the manuscript, and prepared the figures and tables. TS executed the experiments, assisted in data collection, and proofread the manuscript. All authors contributed to the article and approved the submitted version.

## Conflict of Interest

The authors declare that the research was conducted in the absence of any commercial or financial relationships that could be construed as a potential conflict of interest.

## Footnotes

1 http://software.broadinstitute.org/gsea

2 http://baderlab.org/GeneSets

3 http://www.genome.jp/kegg/pathway.html

4 http://www.pantherdb.org/pathway

5 http://geneontology.org

6 http://togows.org

7 http://trap.molgen.mpg.de/cgi-bin/home.cgi
